# Radiographic Markers of Femoroacetabular Impingement: Correlation of Herniation Pit and Femoral Bump with a Positive Cross-Over Ratio

**DOI:** 10.1155/2014/432728

**Published:** 2014-04-27

**Authors:** Max J. Scheyerer, Carol E. Copeland, Jeffrey Stromberg, Thomas Ruckstuhl, Clément M. L. Werner

**Affiliations:** ^1^Department of Surgery, Division of Trauma Surgery, University Hospital Zurich, Raemistrasse 100, 8091 Zurich, Switzerland; ^2^R Adams Cowley Shock Trauma Center, University of Maryland Medical Systems, 22 S. Greene Street, Baltimore, MD 21201, USA

## Abstract

*Introduction*. The goal of this study was to research the association of femoral bumps and herniation pits with the overlap-ratio of the cross-over sign. *Methods*. Pelvic X-rays and CT-scans of 2925 patients with good assessment of the anterior and the posterior acetabular wall and absence of neutral pelvic tilt were enrolled in the investigation. Finally pelvic X-rays were assessed for the presence of a positive cross-over sign, and CT-scans for a femoral bump or a herniation pit. Additionally, if a positive cross-over sign was discovered, the overlap-ratio was calculated. *Results*. A femoral bump was found in 53.3% (*n* = 1559), and a herniation pit in 27.2% (*n* = 796) of all hips. The overlap-ratio correlated positively with the presence of a femoral bump, while a negative correlation between the overlap-ratio and the presence of a herniation pit was found. The latter was significantly more often combined with a femoral bump than without. *Conclusions*. We detected an increased prevalence of femoral bump with increasing overlap-ratios of the cross-over sign indicating a relation to biomechanical stress. The observed decreased prevalence of herniation pits with increasing overlap-ratios could be explained by reduced mechanical stress due to nontightened iliofemoral ligament in the presence of retroversion of the acetabulum.

## 1. Introduction

The subject how far anatomical variations of the hip are responsible for clinical symptoms of femoroacetabular impingement is discussed in literature as well as their influence on the development of osteoarthritis [1–4]. For instance, it is well known that decreased femoral anteversion in combination with decreased acetabular anteversion is a contributor to early osteoarthritis [5].

Two types of impingements with different mechanisms have been described. Cam impingement is the result of a femoral deformity. This is usually a bump at the head-neck junction. Pincer impingement is an impingement based on an acetabular deformity, that is, a deep socket or an acetabular overcoverage.

Reynolds et al. described the cross-over sign (COS) as an indicator for a retroverted acetabulum leading to an overcoverage of the femoral head and consequently to a femoroacetabular impingement [6]. In our study we showed prevalence of 48% within the study population having a minimal overlap of the anterior over the posterior acetabular rim [7].

Like mentioned above, the association between femoral head/neck asphericity and cam type impingement is well documented. Presence of a herniation pit seems to be another indicator for femoroacetabular impingement with described prevalence of 33% in symptomatic hips [1, 8–11].

Up to now all investigations concerning femoroacetabular impingement have been made within symptomatic patients. Hence, it is unknown what degree of overlap (retroversion) can still be considered normal and where the cut-off has to be set indicating risk of impingement.

Based on the association of a femoral bump with a cam type impingement and the high prevalence of herniation pits in affected hips we hypothesize a positive correlation between femoroacetabular impingement with increasing overlap-ratios and the presence of the aforementioned radiologic indicators.

Besides the correlation of herniation pit and femoral bump with gender and increasing age this study was designed to investigate the association of both pathological findings with the overlap-ratios of the cross-over sign. Further, a cut-off value of the overlap-ratio should be determined indicating a pathologic deformity and therefore an increased risk for femoroacetabular impingement.

## 2. Materials and Methods

The study was approved by the Institutional Review Board. Informed consent was not required for this purely radiologic study.

### 2.1. Patients

All patients admitted to the R Adams Cowley Trauma Center between 2000 and 2007 were enrolled in the investigation. Patients who received no anterior-posterior (AP) pelvic radiographs or pelvic CT-scans at the day of admission were excluded. In order to secure no falsification of measurements by malpositioning like mentioned in previous investigations [12], each set of images was validated before inclusion. Further exclusion criteria were obviously traumatic injuries and previous operations like implantation of prostheses. Previous disorders were not excluded. At the end 2925 patients (5928 hips) received an AP-pelvic radiograph and a CT scan, met therefore the requirements, and were evaluated for the presence of positive cross-over sign, femoral bump, and herniation pit.

Prior to the study, four authors trained in reading AP-pelvic radiographs performed a review of the images and pelvic CT-scans.

### 2.2. Validation of Pelvic Radiographs

Rotations of the pelvis in the axial plane have been described to increase the cross-over sign on the side turned away from the source and vice versa. Normal rotation of the pelvis in the axial plane was assumed when the tip of the coccyx was aligned with the middle of the symphysis (within ±5 mm) [13].

Sagittal plane rotations with increased inclination (more of an inlet view) could result in an increased crossing-over while an increased reclination (more of an outlet view) could decrease the cross-over. To minimize falsification of measurements the distance between the sacrococcygeal joint and the symphysis had to be less than 32 mm in men and 47 mm in women [12].

Pelvic rotations in the frontal plane could be corrected electronically with the PACS imaging program and therefore there were no exclusion criteria.

### 2.3. Measurements on Conventional X-Rays

**Figure 1:**
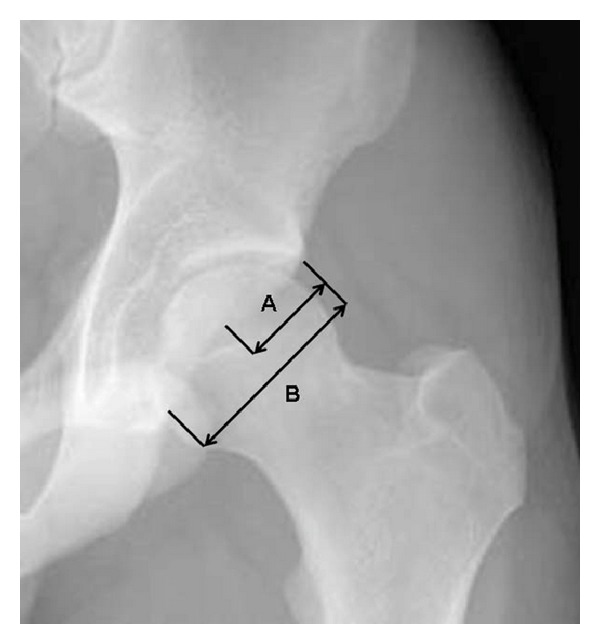
Illustration of the method used to calculate the overlap-ratio A : B. Distance A (extending from the lateral border of the acetabulum to the point where the anterior rim crosses the posterior rim) and distance B (extending from the lateral border of the acetabulum to its posteroinferior border).

Acetabula on AP-pelvic radiographs were evaluated for the presence of the cross-over sign [12]. If present, the overlap-ratio of the anterior over the posterior acetabular rim was calculated using the method described by Siebenrock et al. [12]. Distance A extended from the lateral border of the acetabulum to a point where the anterior rim crosses the posterior rim. Distance B was measured from the lateral border of the acetabulum to its posteroinferior border. The calculated ratio of A : B was called “overlap-ratio of the cross-over sign” (Figure 1).

To measure the overlap-ratio properly, both the anterior and the posterior acetabular walls had to be recognizable and intact. If they were not visible, the concerning acetabulum was excluded.

In the present study, an overlap-ratio of >1% was determined as a positive cross-over sign.

**Figure 2:**
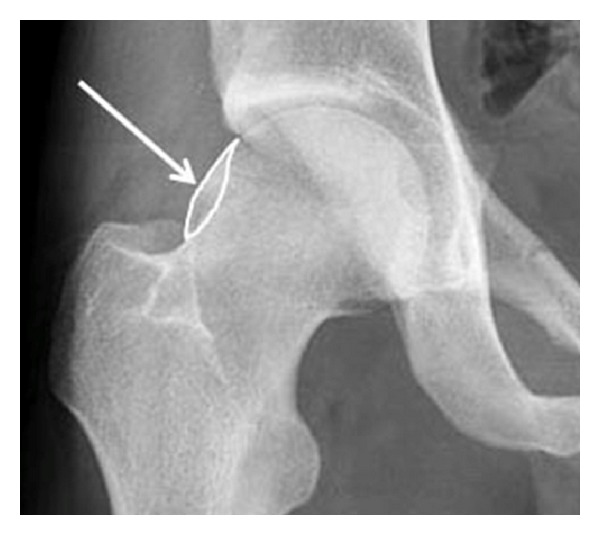
Illustration of a femoral bump seen on a radiograph (arrow, marked area). It is defined as an aspherical part of the femoral head-neck junction.

Additionally, the femoral neck was evaluated subjectively for the presence of a femoral bump like configuration as shown in Figure 2. Femoral bump configuration is defined as an aspherical part of the femoral head-neck junction.

### 2.4. Assessment of Pelvic CT-Scans

**Figure 3:**
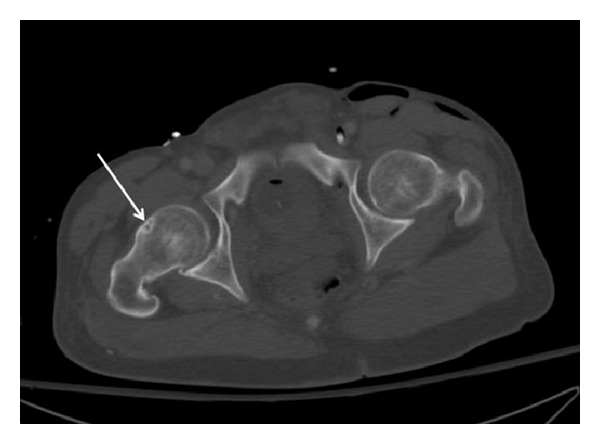
A herniation pit can be seen at the anterosuperior right femoral neck (arrow). It is radiolucencies areas surrounded by a sclerotic margin.

Pelvic CT-scans were evaluated for the presence of a herniation pit at the anterosuperior femoral neck (Figure 3). Herniation pits can be visualized as radiolucency area surrounded by a sclerotic zone.

Since no rotation sensitive measurements were performed it was not necessary to validate the patient's position.

### 2.5. Statistical Methods

Statistical analysis was performed by a statistical consultant using SPSS 13.0 for Windows (SPSS Inc., Chicago, Illinois, USA). Associations between the three binomial variables (presence of femoral bump, crossing-over sign, and herniation pit) were evaluated by means of Pearson's chi-square test. The data were analysed in contingency tables for this purpose. The Mann-Whitney *U* test was applied for detecting significant differences in mean age between the subjects that had a femoral bump and those that did not. This statistical test was also used for the evaluation of age in the herniation pit subgroup compared to the subgroup without herniation pits. A probability value ≤ 0.05 was considered statistically significant.

## 3. Results

### 3.1. Demographic Data

The mean age of the remaining patients was 39.6 years with a range of 14 to 97 years. Included women were significantly older than men (43.2 years versus 38.1 years).

### 3.2. Femoral Bump

Femoral bump was present in 53.3% of all hips. In men (58.4%) it was significantly more prevalent than in women (40.4%) (*P* < 0.001). In contrast, no age-dependency was obvious (*P* = 0.171) (Table 1).

### 3.3. Cross-Over Sign

The mean overlap-ratio was 13.6% (range, 0% to 93%). Statistically significant association between increasing cross-over ratio and presence of a femoral bump could be observed (*P* = 0.032). By contrast, no significant correlation between cross-over sign and presence of a femoral bump exists (*P* = 0.146).

### 3.4. Herniation Pit

Herniation pit was present in 27.2% of all hips. Statistically significant correlation between presence of herniation pits and increasing age (*P* < 0.0001) was obvious. In this context prevalence increased from 7.2% within the age group of 20 to 30 up to 30% in the cohort being 30–40 years old. A maximum could be observed in patients being 50–60 years. Gender specific analysis showed significantly more herniation pits in men (29.8%) than in women (20.3%) (*P* < 0.0001) (Table 1).

Presence of a herniation pit was associated with a positive cross-over sign in 45.0% hips. Increasing overlap-ratios was associated with decreasing prevalence of herniation pits (*P* < 0.0001). Association of herniation pit with a positive cross-over sign was more common in men than in women (48.8% versus 39.1%).

A highly significant correlation was measured between the presence of a herniation pit and the occurrence of a femoral bump (*P* < 0.0001). In this context a herniation pit was found in combination with a femoral bump in 62.1% of all cases (Table 2).

In hips without a herniation pit, a head/neck asphericity was present in 50.1% while in 49.9% the junction was normally configured.

## 4. Discussion

To our knowledge, the current study is the first that evaluated the correlation between the presence of a femoral bump and a herniation pit on one hand and the overlap-ratio of the cross-over sign on the other hand. To provide if crossing-over is present or not a threshold of more than 1% overlap-ratio was determined. Assessment was purely radiographic without taking clinical symptoms into account.

A femoral bump could be found more often in men which is in a line with previous investigations [4]. Further, increasing overlap-ratio of the cross-over sign was associated with higher incidence of femoral bump. To date, the etiology of the femoral bump is still unclear. Jäger et al. hypothesized that a local recruitment of osteoprogenitor cells stimulated by biomechanical forces is responsible for secondary tissue calcification in impingement zones promoting a growing osseous bump deformity [14]. Their hypothesis was supported by the observation that the interval between the onset of symptoms and the diagnosis of a bump deformity was 5.4 years. This finding indicates a mechanical cause for femoral bump as a result of increased contact of the femoral neck with the acetabular rim or labrum in a retroverted acetabulum.

The term herniation pit was initially described by Pitt et al. [15]. The origin of this fibrocystic deformity starts with herniation of soft tissue (collagenous tissue, neocartilage, and reactive new bone) through erosions or perforations on the anterior-superior surface of the femoral neck. It was proposed that the reaction was a result of mechanical, abrasive effects of the overlaying capsule, which is particularly thick. The thickness in this area is due to crossing of circular and vertical fibers, the zona orbicularis, and the lateral part of the iliofemoral ligament. Further contributing factors might be the indirect pressure applied by the overlying straight head of the rectus femoris muscle and the iliopsoas muscle [15–17].

In the current study increasing age was associated with the presence of a herniation pit supporting the hypothesis of stress induced pit formation. We further assumed an increased prevalence of herniation pits with an increasing overlap-ratio as a consequence of direct contact between the femoral neck and the acetabular rim or labrum caused by the overcoverage of the femoral head. But in contradiction to our assumption a negative correlation between the overlap-ratio of the cross-over sign and the occurrence of a herniation pit could be found. Therefore, a direct contact between the femur and the acetabulum is unlikely the cause of pit formation. Searching for an explanation for this surprising finding we reevaluated the mechanism proposed by Pitt et al. [15]. We hypothesize that the version of the acetabulum influences tightness of the iliofemoral ligaments over the femoral neck. An increasing retroversion, and therefore a higher overlap-ratio, places the site of insertion of the ligament more laterally. This leads to a less tightened conduction over the femoral neck. In contrast, a more medial insertion, which is associated with an anteverted acetabulum, would tighten the ligament. Thereby increasing stress is produced and leads to pit formation.

Further, higher incidence of herniation pits in the presence of femoral bump and in men could be shown. In conjunction with the higher incidence of femoral bumps in men the finding could be a possible explanation that more tightened iliofemoral ligament induces formation of herniation pits.

A major limitation of this investigation is the absence of data on the patients' symptoms. A direct identification of symptomatic hips was therefore not possible. As a result we were not able to determine between an asymptomatic hip with a low overlap-ratio and a symptomatic hip with an increased overlap-ratio. Hence, no cut-off value could be calculated. As an arbitrary set threshold we assumed a pathologic deformity in presence of a femoral bump combined with a herniation pit. Whereby appearance of a femoral bump was associated with an increasing overlap-ratio, herniation pits were related to decreased values. These findings made it impossible to find a cut-off value of the overlap-ratio to discriminate between normal and pathologic above all without having data about symptoms.

Another theoretical weakness of this study is the number of four different radiograph-reviewers. To minimize the effects of this confounding factor all of them were carefully instructed by the same physician to evaluate pelvic X-rays and were supervised until being very comfortable with the measurements. In another part of the study [7] they were shown to have an excellent interrater correlation for these measurements.

As a further limiting factor only AP-pelvic radiographs were available to evaluate a femoral bump. Meyer et al. described that the aspherical portion of the femoral head/neck contour might be missed when using AP views [18]. Given that in most cases a femoral bump is detectable on AP-pelvic radiographs assessment was performed very carefully to minimize the effect of this confounding factor. Further, no other standards for estimating femoral bump were used; consequently reliability and validity maybe slightly restricted. However, through the steady supervision we have tried to minimize this limitation.

The detected age difference between men and women was probably the result of inclusion criterion with use of trauma patients for this investigation. It is well-known that young men are at higher risk for accidents than young women, thus attributing to the men's lower mean age.

## 5. Conclusions

In conclusion, increased prevalence of a femoral bump with increasing overlap-ratios of the cross-over sign could be found indicating a relation to biomechanical stress. In contrary, the appearance of a herniation pit was associated with a decreasing overlap-ratio that could be explained with increased tightening of the iliofemoral ligament and the capsule according to the assumption presented by Pitt et al. [15]. Our initial hypothesis was that direct contact between the femoral neck and the acetabular rim induces the formation of a femoral bump and ongoing stress on the femoral neck after the development of a bump leading to growth of a herniation pit could therefore not be confirmed.

As a consequence, our initial aim to find a threshold value for the overlap-ratio to distinguish between a variation from normal anatomy and a pathologic deformity based on the occurrence of a femoral bump and a herniation pit was not possible. Further investigations comparing symptomatic and asymptomatic patients have to be performed to define pathologic overlap-ratios.

## Figures and Tables

**Table 1:** Incidence of femoral bump and herniation pit.

	Overall	Men	Women	Significance of difference (*P*)
Femoral bump	53.3%	58.4%	40.4%	0.000

Herniation pit	27.2%	29.8%	20.3%	0.000
Herniation pit combined with a cross-over sign^†^	45.0%	48.8%	39.1%	

^†^of all hips with a herniation pit.

**Table 2:** Relationship between femoral bump and herniation pit.

	Femoral bump	No femoral bump
With herniation pit	62.1%	37.9%
No herniation pit	50.1%	49.9%
